# What is the burden of respiratory syncytial virus outbreaks in care homes? An enhanced surveillance study in England, winter 2024/25

**DOI:** 10.1017/S0950268826101770

**Published:** 2026-06-15

**Authors:** Luke McGeoch, Kelly Stoker, Megan Rome, Catherine Carey, Andrew Rose, Gayle Dolan, Jackie Cassell, Conall Watson

**Affiliations:** 1 Field Service South-East and London, UK Health Security Agency, UK; 2 Field Epidemiology Training Programme, UK Health Security Agency, UK; 3 Adult Social Care Team, Health Equity & Inclusion Health Division, UK Health Security Agency, UK; 4 Immunisation & Vaccine-Preventable Diseases Division, UK Health Security Agency, UK; 5 North East Health Protection Team, UK Health Security Agency, UK; 6 Centre for Health Service Studies, University of Kent, UK; 7 Brighton and Sussex Medical School, UK; 8 Respiratory Infection Health Protection Research Unit, Imperial College London, UK

**Keywords:** disease outbreaks, homes for the aged, nursing homes, public health surveillance, residential facilities, respiratory syncytial viruses

## Abstract

Care homes are vulnerable to respiratory syncytial virus (RSV) outbreaks, with residents at risk of severe disease outcomes, though surveillance studies are lacking. In September 2024, RSV vaccination was introduced for adults in England aged 75–79 years, excluding most residents (median age 86 years). Between October 2024 and March 2025, we prospectively extracted information on RSV outbreaks in English care homes reported to UK Health Security Agency (UKHSA). Enhanced surveillance questionnaires (ESQs) were completed for a sample of outbreaks, including information on symptomatic residents, testing, and outcomes. Acute respiratory infection (ARI) cases were defined symptomatically, RSV cases based on test-positive RSV (≤5 residents tested per home), and RSV outbreaks as ARI outbreaks including ≥1 RSV case. We described outbreak trends, attack rates, hospitalizations, and deaths. Of 2419 ARI outbreaks in care homes, 222 (9%) were RSV outbreaks, with additional respiratory viruses identified in 69 (31%). Among 48 (22%) RSV outbreaks completing ESQs, the median ARI attack rate was 15% (IQR 10%–24%). Of 350 ARI cases, 32% (112) were confirmed RSV cases on routine testing, 20% (71) were hospitalized, and 6% (20) died. These findings demonstrate the vulnerability of care home residents to RSV infection, hospitalization, and death, which are potentially preventable through vaccination.

## Key results

- Between October 2024 and March 2025, RSV was detected in 9% (*n* = 222) of ARI outbreaks in care homes.
- Among 48 outbreaks included in enhanced surveillance, the median resident attack rate for ARI was 15%.
- Among 350 care home resident ARI cases in RSV outbreaks, 20% were hospitalized and 6% died.

## Introduction

Respiratory syncytial virus (RSV) is a common cause of acute respiratory infection (ARI), with infections occurring year-round but demonstrating a mid-winter peak in England between October and February each year [1]. Among older adults, high rates of RSV-associated hospitalization and mortality are similar to those observed for influenza- and SARS-CoV-2-associated ARI [2–7]. This disease burden is typically underestimated by routine surveillance systems [2]. Care homes are residential settings that provide personal and/or nursing care to individuals. The majority of care homes in England are privately owned and operated, though over 60% of care home residents are publicly funded [8]. In October 2024, 344348 individuals were residing in 10962 registered older adult care homes in England [9]. Previous studies have demonstrated a high risk of RSV infection, outbreaks, and severe disease outcomes in older adult care settings, though national surveillance studies of RSV outbreaks in care homes are lacking [10–12]. Frequent interactions between residents, staff, healthcare professionals, visitors, and external service providers increase the risk of ARI outbreaks in care homes [10, 11, 13, 14]. Furthermore, the high prevalence of chronic diseases, clinical frailty, and immunosenescence among residents increases the risk of severe RSV disease [2, 12, 14–16].

In September 2024, England introduced bivalent pre-F RSV vaccine (Abrysvo, Pfizer) for adults aged 75–79 years, following Joint Committee on Vaccination and Immunisation (JCVI) advice [17]. Subsequently, vaccine effectiveness against hospitalization for RSV-associated ARI in this group was estimated to be 82%, and 87% in those with severe disease [17]. Adults aged 80 years and older were not included in the initial programme due to insufficient data on vaccine efficacy in this age group. The median age of residents in older adult care homes in England is 86 years [8], making most residents ineligible for RSV vaccination.

We aimed to describe the frequency and severity of RSV outbreaks in care homes in England during winter 2024–2025, to inform future RSV vaccination policy in these settings by providing evidence on the burden and impact of infection.

## Methods

### Study design and setting

A prospective enhanced surveillance study of RSV outbreaks in registered care homes in England was conducted between 14 October 2024 and 9 March 2025.

### Definitions

Study definitions, shown in Table 1, were selected for consistency with UK Health Security Agency (UKHSA) guidance on management of ARI outbreaks in care homes [1]. For the purposes of identifying recurrent outbreaks occurring in the same setting, outbreaks were defined as ending five days after symptom onset in the last symptomatic resident [1].

**Table 1.** Study definitions Table 1. long description.

Category	Metric	Definition
Symptoms	Acute respiratory infection (ARI)	Acute onset of one or more of the following symptoms and a clinician’s judgement that the illness is due to an ARI: runny nose, sore throat, cough, wheeze.
	Influenza-like illness (ILI)	Oral or tympanic temperature of at least 37.8 °C and acute onset of at least one of the following symptoms: cough, sore throat, coryza, dyspnea, hoarseness, sneezing, wheezing. Alternatively, an acute deterioration in physical or mental ability without other known cause.
Cases	ARI case	A care home resident demonstrating ARI or ILI symptoms during an RSV outbreak.
	RSV case	An ARI case with a polymerase chain reaction or point-of-care test detection of RSV.
Outbreaks	ARI outbreak	A care home with ≥2 ARI cases with symptom onset dates within 5 days of one another.
	RSV outbreak	ARI outbreak with ≥1 confirmed RSV case.

### Data sources

ARI outbreaks in care homes in England are voluntarily reported to UKHSA Health Protection Teams [1]. Up to five symptomatic residents in the care home may be tested for RSV and other respiratory viruses for outbreak confirmation using nose and throat swabs and polymerase chain reaction (PCR) testing at a public health laboratory. Diagnostic virological testing may be conducted for individuals admitted to hospital. Public health testing may not be done where influenza is strongly suspected as the source of the outbreak and is being managed empirically, or if clinical testing of admitted patients is considered to have provided adequate information [1]. Weekly extraction of ARI outbreaks in care homes, based on the date of report of the outbreak, was performed using the UKHSA Case and Incident Management System. The extracted ARI outbreak records were manually reviewed, and RSV named as a causative organism where confirmation of a positive RSV test result was documented. Where positive test results were documented for multiple respiratory viruses, all were named equally as causative organisms.

An enhanced surveillance questionnaire (ESQ) was completed with care home managers via telephone 30 days after the date of outbreak onset to capture both incident-level and case-level information. Initially, ESQ completion was attempted with all care homes reporting RSV outbreaks between weeks 44 (4 November 2024) and 48 (8 December 2024) of 2024. Between week 49 of 2024 (9 December 2024) and week 9 of 2025 (9 March 2025), due to a substantial increase in reported care home RSV outbreaks compared to the previous season, a pragmatic decision was taken to attempt ESQ completion with a random sample of 25% of RSV outbreaks reported each week, rounding up where necessary. Incident-level information included the total number of residents and staff at the care home, the number of residents eligible for RSV vaccination and whether vaccination had been offered in the care home, the number of resident ARI cases and confirmed RSV cases, and the number of hospital admissions and deaths from any cause among resident ARI cases within 30 days of outbreak onset, defined as the date of symptom onset in the first case. Case-level information collected for ARI cases included symptom onset date, RSV test result status, age at outbreak onset, chronic diseases linked to RSV disease severity (asthma, cancer, chronic heart disease, chronic kidney disease, chronic non-asthmatic pulmonary disease, dementia, diabetes, stroke, or transient ischaemic attack), and hospital admission and discharge dates. Where case-level information was not available during the initial telephone interview with care home managers, follow-up was attempted to obtain missing data.

### Data analysis

We calculated the proportion of all ARI outbreaks in care homes that were RSV outbreaks and described the frequencies of RSV outbreaks by reporting week and geographical region. For RSV outbreaks with ESQs completed, including mixed pathogen outbreaks, we calculated attack rates for ARI and RSV (the percentage of care home residents meeting the case definitions for ARI and RSV, respectively) and the percentage of resident ARI cases with all-cause hospital admissions and deaths. Mean and median estimates were computed for all care homes combined. These metrics were also compared between RSV outbreaks in which RSV was the only respiratory virus identified on pathogen testing and those with at least one other pathogen detected. For outbreaks with case-level data provided in ESQs, we calculated the median age of cases and the percentage of cases above and below RSV vaccination age, the frequency and percentage of comorbidities reported, the proportion of residents admitted to hospital who died, and the median length of hospital stay among those discharged from hospital. All analyses were conducted using R version 4.4.0 [18].

## Results

### Total RSV outbreaks

**Figure 1.**
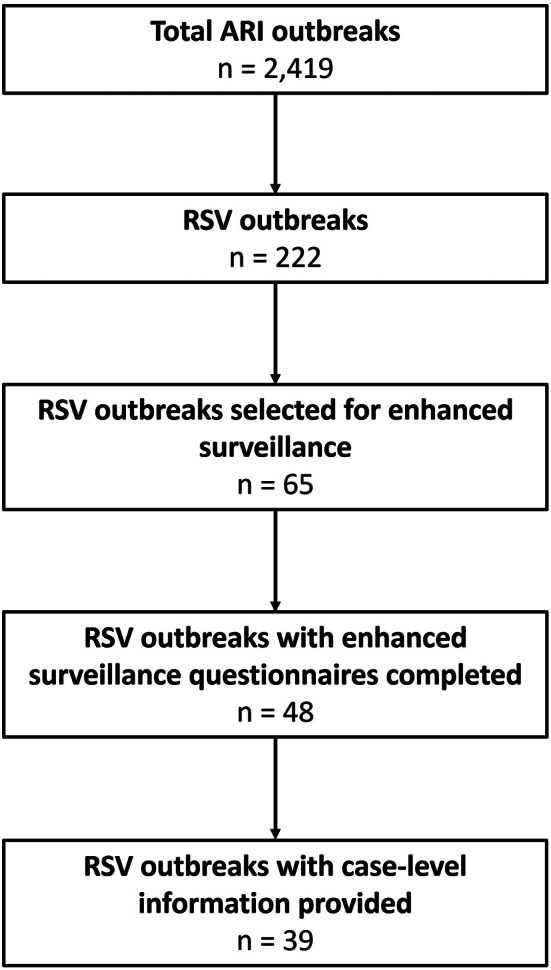
Flow chart showing RSV outbreaks and cases included in the study. Figure 1. long description.

**Figure 2.**
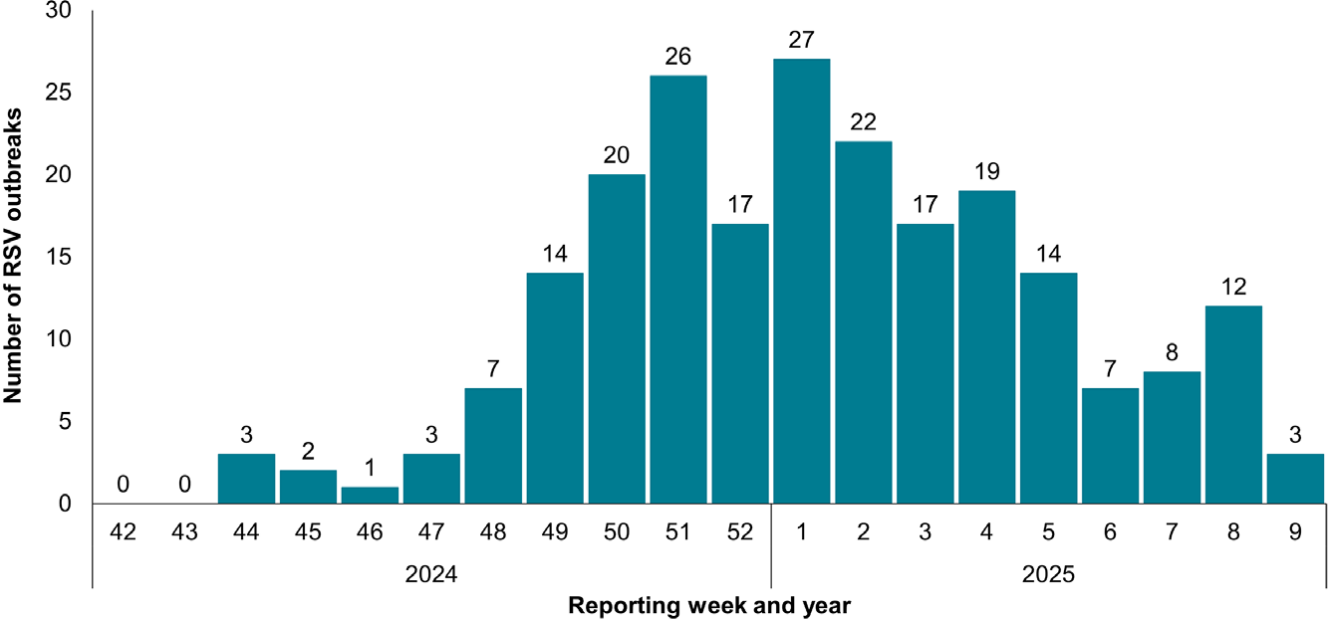
RSV outbreaks in care homes in England by reporting week, 14 October 2024 – 9 March 2025. Figure 2. long description.

Between 14 October 2024 (week 42 2024) and 9 March 2025 (week 9 2025), there were 2419 ARI outbreaks reported in care homes in England. Of these, 222 (9.2%) reported at least one positive RSV test result (Figures 1 and 2). In 69 (31%) of these RSV outbreaks, additional respiratory pathogens were identified (Table 2), most commonly influenza (*n* = 37), rhinovirus (*n* = 14), or SARS-CoV-2 (*n* = 9).

**Table 2.** Number of RSV outbreaks in care homes in England with other respiratory viruses detected, 14 October 2024–9 March 2025 Table 2. long description.

Organism	Number of outbreaks	Percentage
Total RSV outbreaks	222	100.0
At least one other respiratory virus	69	31.1
Influenza virus	37	16.7
Rhinovirus	14	6.3
SARS-CoV–2	9	4.1
Picornavirus	7	3.2
Human metapneumovirus	5	2.3
Seasonal coronavirus	3	1.4
Parainfluenza virus	3	1.4
Enterovirus	1	0.5

### Enhanced surveillance

There were 16 RSV outbreaks reported between weeks 44 and 48 of 2024, of which ESQs were completed for 12 (75.0%). There were 206 outbreaks reported between week 49 of 2024 and week 9 of 2025, with 49 sampled for follow-up; of these, ESQs were completed for 36 (73.5%). Overall, ESQs were attempted for 65 RSV outbreaks and completed for 48 (73.8%) outbreaks. Two outbreaks occurred in the same care home, therefore, 47 care homes were included in the study, supporting 2032 residents (median 39 per care home, IQR 28–55, range 17–159) and employing 2840 staff members (median 54 per care home, IQR 38–75, range 12–159). Forty-four outbreaks reported on whether RSV vaccination had been offered to eligible residents prior to outbreak onset, with 16 (36.4%) stating a vaccination visit had taken place from a healthcare provider. Thirty-four outbreaks reported the number of residents eligible for RSV vaccination, with a median of 10% of residents being eligible (IQR 7–12%, range 0–28%).

In total, 350 (16.8%) residents met the ARI case definition during RSV outbreaks. Of these, there were 112 (32.0%) confirmed RSV cases, 71 (20.3%) hospital admissions, and 20 (5.7%) deaths from any cause within 30 days of outbreak onset. Median attack rates among residents were 15.3% (IQR 9.9–23.5%, mean 19.0%, range 3.8–52.6%) for ARI and 5.4% (IQR 3.3–8.5%, mean 6.4%, range 1.3–22.2%) for confirmed RSV. A median of 20.0% (IQR 0–38.1%, mean 25.4%, range 0–100.0%) of cases were admitted to hospital and a median of 0% (IQR 0–9.6%, mean 5.7%, range 0–40.0%) died.

Table 3 compares the characteristics of RSV-only outbreaks (*n* = 34) and mixed pathogen outbreaks (*n* = 14). Care homes reporting both types of outbreak had similar resident population sizes. The median ARI attack rate was higher in mixed pathogen outbreaks, whereas the median RSV attack rate and percentage of ARI cases hospitalized were higher in RSV-only outbreaks.

**Table 3.** Attack rates, hospitalizations, and deaths in RSV-only versus mixed pathogen outbreaks with ESQs completed Table 3. long description.

	RSV-only (*n* = 34)	Mixed (*n* = 14)
**Median number of residents (IQR)**	40.0 (29.0–55.0)	36.5 (25.2–51.0)
**ARI attack rate**		
Median (IQR)	14.3% (9.4–17.7)	21.1% (15.3–29.0)
Mean (SD)	17.2% (12.3)	23.3% (13.7)
**RSV attack rate**		
Median (IQR)	5.8% (3.4–8.6)	4.0% (2.7–7.6)
Mean (SD)	6.4% (4.1)	6.4% (5.9)
**Percentage of ARI cases hospitalized**		
Median (IQR)	20.0% (7.3–40.0)	4.2% (0–22.3)
Mean (SD)	28.7% (28.7)	17.4% (25.7)
**Percentage of ARI cases who died**		
Median (IQR)	0% (0–12.2)	0% (0–0)
Mean (SD)	6.5% (10.8)	3.7% (8.3)

At least partial case-level information was provided for 39 (81.3%) outbreaks and 226 (64.6%) resident ARI cases. Among 221 resident cases with age reported, median age was 87 years (IQR 82–91, range 34–101); 182 (82.4%) cases were above the age threshold for RSV vaccination in England and 22 (10.0%) were below the age threshold. Chronic disease information was provided for 35 outbreaks (Table 4), with dementia being the most commonly reported condition (63.2%) followed by diabetes (21.6%) and chronic heart disease (21.1%).

**Table 4.** Frequency of chronic diseases among resident RSV cases with data available (*n* = 206) reported in outbreaks with ESQs completed, 14 October 2024–2 March 2025 Table 4. long description.

Chronic disease	Number of cases	Percentage
Any chronic disease	179	87.0
Dementia	129	63.2
Diabetes	44	21.6
Chronic heart disease	43	21.1
Cancer	29	14.2
Chronic kidney disease	27	13.2
COPD or other non-asthmatic chronic lung disease	27	13.2
Asthma	22	10.8
Stroke or TIA	17	8.3

Among the 49 (69.0%) hospitalized ARI cases with case-level information recorded, 8 (16.3%) died from any cause within 30 days of outbreak onset, and the median duration of hospital admission among those discharged back to the care home (data available for 41 cases) was 5.0 days (IQR 2.8–8.3, mean 6.8, range 0–25).

## Discussion

Our enhanced surveillance indicates the importance of RSV as an outbreak pathogen in older adult care homes. During winter 2024–2025, RSV was detected in 222 ARI outbreaks in care homes in England, 9% of all those reported to UKHSA Health Protection Teams. RSV outbreaks peaked in December and January. Enhanced surveillance data collected for 48 RSV outbreaks demonstrated a median attack rate of 15% for ARI, with 20% of cases being hospitalized and 6% dying from any cause within 30 days of outbreak onset. Ten per cent of residents and eight per cent of ARI cases in care homes with RSV outbreaks were eligible for RSV vaccination.

Older adult care home residents have generally poorer health than older adults living in the community, and the survival gap between these two groups has increased over time in England [19], as has the prevalence of severe disability and complex multimorbidity among care home residents [20]. Care home residents are known to be susceptible to a range of respiratory infections, with frequent interactions with other residents, staff members, and visitors supporting ingress and transmission [10, 11, 13, 14].

Previous studies have demonstrated a higher risk of RSV infection, hospitalization, and mortality among care home residents compared to community-dwelling adults of the same age, likely reflecting a combination of frailty, comorbidity, and immunosenescence [2, 12, 14–16, 21, 22]. An observed attack rate of 15% for ARI cases during RSV outbreaks in the current study is consistent with attack rates of 7–48% for laboratory-confirmed RSV reported elsewhere [12]. Our finding that 20% of ARI cases were hospitalized is also comparable to figures of 10–23% reported in previous studies, whilst a case fatality ratio of 6% is slightly lower than previously reported rates of 8–23% [12]. However, previous studies were limited to inclusion of a smaller number of care homes, often single institutions in the case of outbreak studies, and had varying methodologies [11, 12]. Among studies reporting on RSV infections in community-dwelling older adults, hospitalization and case fatality ratios of 24% and 8%, respectively, have been described [21]. The burden of RSV infections and severe disease outcomes would be expected to vary between winter seasons and between geographies based on differences in the nature (size, resident, and staff population) of care homes; definitions, reporting, and management of RSV cases and outbreaks; testing policies and modalities; timing of outbreaks and studies; and study methodologies.

During winter 2024–2025, influenza was detected in 1169 outbreaks in care homes in England [23]. This is consistent with a greater preponderance of published studies reporting influenza rather than RSV as the cause of outbreaks identified in long-term care facilities internationally, though median attack and case fatality rates were found to be higher where RSV was reported as the cause of the outbreak [24]. More recent published studies describing confirmed influenza outbreaks in care homes in Northern Ireland and one region of England reported average attack rates of around 25% among residents [25, 26]. Among hospitalized adults more widely, clinical outcomes and mortality are similar for ARI associated with RSV and influenza [3, 7].

In addition to causing morbidity and mortality, by causing illness in staff members, RSV outbreaks may adversely affect the quality of life of care home residents by impacting staff availability and the ability to be looked after by familiar staff. Whilst it is recommended that care homes should remain open to safely managed visiting during outbreaks [1], in practice, some visiting is likely to be avoided, reducing quality of life both for visitor and resident and potentially reducing safeguarding opportunities. Additionally, communal activities may be cancelled or postponed. Further, RSV outbreaks impact on service delivery by care homes and incur substantial healthcare demand and costs [7, 14].

Most residents and ARI cases identified in this study did not meet contemporaneous age-based eligibility for RSV vaccination. Others have observed that the frequency of severe RSV disease outcomes increases with age among older adults [7, 14, 27]. Evidence from older adult RSV vaccination programmes in the USA has demonstrated that RSV vaccines have similar effectiveness against severe disease outcomes in those aged ≥75 or ≥80 years compared to younger adults [28–30]. RSV vaccine coverage among eligible older adults in England reached 61.7% in October 2025 [1], whilst influenza vaccine coverage during the 2024–2025 winter season was 74.9% among all adults aged ≥65 years and 74.1% among residents of older adult care homes [23, 31].

The temporal distribution of RSV outbreaks we observed is consistent with other surveillance systems demonstrating later RSV activity in older adults compared to children [1], and may also reflect delays in outbreak reporting.

A key strength of this study was its coverage of care homes across England, and characterization of the age and health status of affected residents in sampled homes. Timely collection of data through interviewer-led questionnaires supported information recall. This study also had several limitations. Overall data on ARI outbreaks are affected by differences in reporting practices between care homes and regions. The availability and implementation of RSV testing, including the threshold for testing symptomatic residents, also varies between care homes. Further, the absence of universal testing artificially constrained detections of confirmed RSV cases. Though we reported on detections of mixed infection outbreaks, it was not possible to ascertain which pathogen was the predominant driver of these outbreaks. Additionally, symptoms in ARI cases could have been caused by other organisms or aetiologies. RSV cases and outbreaks may have been underascertained where care homes detected SARS-CoV-2 through lateral flow device testing and did not undertake further pathogen testing, as well as potential lower sensitivity of PCR for respiratory viruses among older adults [27, 32]. Data collection was dependent on reporting by care home managers without additional verification through medical records or death certificates. Limited information was available for staff members, since testing is not routinely conducted for these individuals; staff illness and absence could therefore be characterized in further studies. Finally, case-level data were not reported for all outbreaks.

## Conclusion

This national enhanced surveillance study provides a real-world insight into the frequency and severity of RSV outbreaks within care home settings, highlighting the vulnerability of residents living in these settings to infection and severe disease outcomes. Our results were presented to the UK Joint Committee on Vaccination and Immunisation, supporting the committee’s advice on extending RSV vaccination to all residents of care homes for older adults [31]. Our findings also highlight the importance of enhanced surveillance to better monitor and respond to ARI outbreaks in these high-risk environments, and to monitor the impact of vaccination on disease burden and severity.

## Data Availability

Any queries regarding data availability can be directed to the authors, who will consider reasonable requests for access to anonymized data in line with the UKHSA data release policy.

## Long descriptions

### Table 1 Long description

RSV outbreak: an ARI outbreak with at least one confirmed RSV case.

ARI outbreak: ≥2 ARI cases in a care home within 5 days of each other.

Outbreaks:

RSV case: an ARI case with laboratory-confirmed RSV (PCR or point-of-care test).

ARI case: any care home resident with ARI or ILI symptoms during an RSV outbreak.

Cases:

Influenza-like illness (ILI): fever ≥37.8°C plus at least one respiratory symptom (e.g., cough, sore throat), or acute deterioration without another cause.

Acute respiratory infection (ARI): acute onset of at least one symptom (runny nose, sore throat, cough, or wheeze) with clinical judgement of ARI.

Symptoms:

This table presents definitions grouped into three categories: symptoms, cases, and outbreaks.

Navigate back to Table 1.

### [Figure 1] Long description

The diagram shows progressive reduction in the number of outbreaks included at each stage, from all reported acute respiratory infection outbreaks to a final subset with detailed case-level data.

The final box reads: “RSV outbreaks with case-level information provided, n = 39.”

The fourth box reads: “RSV outbreaks with enhanced surveillance questionnaires completed, n = 48.”

The third box reads: “RSV outbreaks selected for enhanced surveillance, n = 65.”

An arrow leads to the second box: “RSV outbreaks, n = 222.”

The top box reads: “Total ARI outbreaks, n = 2,419.”

This figure is a vertical flow diagram consisting of five stacked rectangular boxes connected by downward arrows, illustrating the selection of outbreaks included in the study.

Navigate back to [Figure 1].

### Figure 2 Long description

Overall, the figure shows a clear winter peak in RSV outbreaks, with highest activity in late December and early January.

Outbreaks are absent in weeks 42–43, then remain low through week 47 (1–3 per week). Counts rise from week 48 (7) to week 51 (26). After a slight dip in week 52 (17), the peak occurs in week 1 of 2025 (27), followed by week 2 (22). Numbers then decline overall, with minor fluctuations: week 4 rises slightly (19) and week 8 shows a small rebound (12), before dropping to 3 by week 9.

Vertical bar chart showing weekly counts of RSV outbreaks in care homes in England from week 42 of 2024 to week 9 of 2025. The x-axis displays reporting weeks (42–52 for 2024, then 1–9 for 2025), and the y-axis shows the number of outbreaks (0–30).

Navigate back to Figure 2.

### Table 2 Long description

Enterovirus: 1 (0.5%)

Parainfluenza virus: 3 (1.4%)

Seasonal coronavirus: 3 (1.4%)

Human metapneumovirus: 5 (2.3%)

Picornavirus: 7 (3.2%)

SARS-CoV-2: 9 (4.1%)

Rhinovirus: 14 (6.3%)

Influenza virus: 37 outbreaks (16.7%)

Breakdown of co-detected viruses:

Outbreaks with at least one additional virus: 69 (31.1%).

Total RSV outbreaks: 222 (100%).

This table summarises how often additional respiratory viruses were detected alongside RSV in 222 care home outbreaks.

Navigate back to Table 2.

### Table 3 Long description

Mixed mean: 3.7%

RSV-only mean: 6.5%

Median is 0% in both groups:

Mortality (percentage of ARI cases):

Mixed: median 4.2% (mean 17.4%)

RSV-only: median 20.0% (mean 28.7%)

Hospitalisation (percentage of ARI cases):

Mean is equal (6.4%) in both groups

Mixed: median 4.0%

RSV-only: median 5.8%

RSV attack rate:

Mixed: median 21.1% (mean 23.3%)

RSV-only: median 14.3% (mean 17.2%)

ARI attack rate:

Mixed: 36.5 (IQR 25.2–51.0)

RSV-only: 40 (IQR 29.0–55.0)

Care home size:

Mixed pathogen outbreaks (n = 14)

RSV-only outbreaks (n = 34)

This table compares characteristics of two groups of outbreaks with enhanced surveillance data:

Navigate back to Table 3.

### Table 4 Long description

Stroke or transient ischaemic attack: 17 (8.3%)

Asthma: 22 (10.8%)

Chronic lung disease (including COPD): 27 (13.2%)

Chronic kidney disease: 27 (13.2%)

Cancer: 29 (14.2%)

Chronic heart disease: 43 (21.1%)

Diabetes: 44 (21.6%)

Dementia: 129 (63.2%)

Any chronic disease: 179 cases (87.0%)

This table describes comorbidities among 206 resident RSV cases with available data.

Navigate back to Table 4.
